# Current misconception 3: that subgroup-specific trial mortality results often provide a good basis for individualising patient care

**DOI:** 10.1038/bjc.2011.79

**Published:** 2011-03-29

**Authors:** R Peto

**Affiliations:** 1 Clinical Trial Service Unit and Epidemiological Studies Unit (CTSU), Richard Doll Building, Old Road Campus, Oxford OX3 7LF, UK

## Abstract

Misconceptions and ill-founded theories can arise in all areas of science. However, the apparent accessibility of many epidemiology findings and popular interest in the subject can lead to additional misunderstandings. The article below is the third in an occasional series of short editorials highlighting some current misinterpretations of epidemiological findings. Invited authors will be given wide scope in judging the prevalence of the misconception under discussion. We hope that this series will prove instructive to cancer researchers in other disciplines as well as to students of epidemiology. *Adrian L Harris* and *Leo Kinlen*

For breast cancer, as for many other diseases, treatment that is appropriate for one patient may be inappropriate for another. Ideally, therefore, what is wanted from trials is not only an answer to the question ‘Is this treatment helpful on average for a wide range of patients?’, but also an answer to the question ‘For which recognisable categories of patient is this treatment particularly helpful?’ (EBCTCG, 2005a,2005b)

In general, however, this ideal cannot be achieved directly from subgroup-specific analyses of clinical trial results because apparent differences between the proportional risk reductions in different subgroups of the patients in a trial (or even in a meta-analysis of many trials) are often surprisingly unreliable. For example, even if the proportional effects of the trial treatment in specific subgroups really are importantly different, standard subgroup analyses are so insensitive that they may well fail to demonstrate these real differences. Conversely, even if the trial results suggest that the trial treatment works in some subgroups but not in others (thereby giving the appearance of a ‘qualitative interaction’), this may still not be good evidence for subgroup-specific treatment preferences. The play of chance often produces qualitatively wrong answers in particular subgroups in trials (or in meta-analyses of trials) that could, if interpreted incautiously, lead to millions of people being treated inappropriately or untreated inappropriately.

Questions about such ‘interactions’ between patient characteristics and the effects of treatment are easy to ask, but are surprisingly difficult to answer reliably. Apparent interactions can often be produced by the play of chance and, in particular subgroups, can mimic or obscure some of the moderate treatment effects that might realistically be expected. To illustrate this, a subgroup analysis was performed based on the astrological birth signs of 17 000 patients in the Second International Study of Infarct Survival (ISIS-2), a randomised trial of 1 month of daily aspirin *vs* placebo for suspected acute myocardial infarction. Overall in this trial, the 1-month survival advantage produced by aspirin was demonstrated conclusively (804 vascular deaths among 8587 patients allocated aspirin *vs* 1016 among 8600 allocated no aspirin; 23% proportional reduction, *P*<0.000001). To demonstrate the unreliability of subgroup analyses, these findings were subdivided into 12 subgroups according to the patients’ medieval astrological birth signs; the results in each were examined to find which of the 12 appeared least promising, and when just those with apparently unpromising results were collected together, it was ‘discovered’ that aspirin appeared totally ineffective for patients born under Libra or Gemini (Table 1) (ISIS, 1988)!

It would be unwise to conclude from such a result that patients born under the astrological birth sign of Libra or Gemini should not be given aspirin if they have a heart attack. However, similar conclusions based on ‘exploratory’ data-derived subgroup analyses, which from a purely statistical viewpoint are no more reliable than these astrological subgroup analyses, are often reported and believed, with inappropriate effects on worldwide clinical practice.

There are three partial remedies for this unavoidable conflict between the reliable subgroup-specific conclusions that doctors and patients want and need, and the statistically unreliable findings that direct subgroup-specific analyses can usually offer. However, the extent to which these remedies are helpful in particular instances is one on which informed judgements differ.

First, where there are good prior reasons for anticipating that the proportional effects of treatment might be very different in different circumstances, one particular subgroup analysis may be prespecified in the study protocol, along with a prediction of the direction of the proposed interaction. (For example, it was expected that the benefits of fibrinolytic therapy for acute myocardial infarction would be greater the earlier such patients were treated, and so some studies prespecified that the statistical analyses would be subdivided by the number of hours from the onset of symptoms to treatment: FTT, 1994.) Although a single prespecified subgroup-specific analysis can then be taken somewhat more seriously than other subgroup analyses, protocols that pre-specify several subgroup analyses as ‘secondary outcomes’ can yield importantly wrong answers.

The second approach is to take the proportional risk reduction that is suggested by the overall results of the trial (or, better still, by the overall results from a meta-analysis of all such trials) as a semi-quantitative guide to the proportional risk reductions in various specific subgroups of patients, giving little weight to the apparent results in each of such subgroups. This is clearly the right way to interpret the astrological ‘findings’ in Table 1, and, if used sensibly, may also in many other circumstances provide the best guide as to whether one treatment is better than another in particular subgroups.

The main determinant of whether toxic or expensive treatment is worthwhile is the absolute risk reduction that it produces, and it is perfectly proper to use the fact that patients who already have a very good prognosis anyway and are at low absolute risk cannot have a large absolute benefit (for, even if a small risk is halved the absolute benefit is small). Classification of patients as being at low (or high) risk of an adverse outcome is often a useful guide as to which patients can expect a small (or large) absolute gain. Appropriate clinical use of this low-risk/high-risk split may not require support from formal subgroup analyses – indeed, it could even be damaged by incautious reliance on such analyses.

The third approach is to be influenced, in discussing the likely effects on mortality in specific subgroups of breast cancer patients, not only by mortality analyses but also by analyses of recurrence, early recurrence, local recurrence or some other major ‘surrogate’ outcome. For, if the overall results are similar but much more highly significant for recurrence than for mortality, subgroup analyses with respect to the former may be more stable and may provide a better guide as to whether there are any major differences between subgroups in the proportional risk reduction produced by treatment (EBCTCG, 2005a,2005b).

The appropriate interpretation of apparently different results in different subgroups of trial results is still one of the most difficult matters of judgement in the interpretation of randomised evidence; at present, many clinicians and regulatory agencies pay far too much attention to irregularities between the apparent effects in different subgroups, to the potential detriment of the care of individual patients.

## Figures and Tables

**Table 1:** False-negative mortality effect in a subgroup defined only by the medieval astrological birth sign: the ISIS-2 trial of aspirin among over 17 000 patients with acute myocardial infarction

**Astrological birth sign**	**No. of 1-month deaths (aspirin *vs* placebo)**	**Statistical significance**
Libra or Gemini	150 *vs* 147	NS
All other signs	654 *vs* 869	2*P*<0.000001
Any birth sign^a^	804 (9.4%) *vs* 1016 (11.8%)	2*P*<0.000001

a Appropriate overall analysis for assessing the true effect in all subgroups. Astrology divides birth dates into 12 ‘birth signs’ (which depend only on the day and month of birth, not the year of birth). To demonstrate the potential unreliability of subgroup analyses, the ISIS-2 patients were divided into 12 subgroups according to their astrological birth sign, and the apparent effects of aspirin were calculated separately in each of these 12 subgroups. Because of the play of chance, the apparent effects differed from one subgroup to another, ranging from no apparent effect of aspirin in two subgroups (Libra and Gemini) to aspirin apparently halving the mortality in another (Capricorn).
